# Effect and mechanism of GLP-1 on cognitive function in diabetes mellitus

**DOI:** 10.3389/fnins.2025.1537898

**Published:** 2025-03-18

**Authors:** Xiaoke Dou, Lei Zhao, Jing Li, Yaqiu Jiang

**Affiliations:** ^1^Department of Endocrinology and Metabolism, The First Hospital of China Medical University, Shenyang, China; ^2^Department of Gerontology, The First Hospital of China Medical University, Shenyang, China; ^3^Department of Laboratory Medicine, National Clinical Research Center for Laboratory Medicine, The First Hospital of China Medical University, China Medical University, Shenyang, China

**Keywords:** glucagon-like peptide-1, cognitive impairment, diabetes mellitus, mechanism, neuroprotection

## Abstract

**Background:**

Diabetes mellitus (DM) is a metabolic disorder associated with cognitive impairment. Glucagon-like peptide-1 (GLP-1) and its receptor (GLP-1R) have shown neuroprotective effects.

**Scope of review:**

This review explores the impact of DM on cognitive function. Diabetes-related cognitive impairment is divided into three stages: diabetes-associated cognitive decrements, mild cognitive impairment (MCI), and dementia. GLP-1R agonists (GLP-1RAs) have many functions, such as neuroprotection, inhibiting infection, and metabolic regulation, and show good application prospects in improving cognitive function. The mechanisms of GLP-1RAs neuroprotection may be interconnected, warranting further investigation. Understanding these mechanisms could lead to targeted treatments for diabetes-related cognitive dysfunction.

**Major conclusions:**

Therefore, this paper reviewed the regulatory effects of GLP-1 on cognitive dysfunction and its possible mechanism. Further research is required to fully explore the potential of GLP-1 and its analogs in this context.

## 1 Introduction

Diabetes mellitus (DM), characterized by chronic hyperglycemia, is escalating globally, contributing to heightened morbidity and mortality from diabetic complications (Forbes and Cooper, 2013; Kirvalidze et al., 2022; Yaribeygi et al., 2021). About 1 in 11 adults worldwide have diabetes mellitus, 90% of whom have type 2 diabetes mellitus(T2DM) (Zheng et al., 2018). The latest International Diabetes Federation (IDF) report indicates a 10.5% prevalence of T2DM in adults in 2021, projected to reach 12.2% by 2045 (Sun et al., 2022; Yan et al., 2022). T2DM has been associated with cognitive impairment, emphasizing its role as a risk factor for cognitive impairment (Pelle et al., 2023; You et al., 2021). Glucagon-like peptide-1 (GLP-1) receptor agonists (GLP-1 RAs), as hypoglycemic drugs, has attracted more and more attention in cognitive function and protective effect of neurons.

GLP-1 is an endogenous hormone with 30 amino acids that is mainly derived from the preproglucagon molecule in enteroendocrine L-cells of the gastrointestinal tract (Bae and Song, 2017; Yaribeygi et al., 2021). However, endogenous GLP-1 is also produced by preproglucagon (PPG) neurons in the brain (Brierley et al., 2021; Holt et al., 2019; Müller et al., 2019). GLP-1 receptor (GLP-1R) is widely distributed in the central nervous system, mainly in neurons (Hayes, 2012; Vrang and Larsen, 2010), with high expression in glutamatergic neurons of the area postrema (AP) and nucleus of the solitary tract (NTS) (Ludwig et al., 2021a; Ludwig et al., 2021b). In recent years, it is believed that GLP-1 and its analogs can affect central nervous system functions such as cognition and neuroprotection (Hunter and Hölscher, 2012; McClean et al., 2011; Yaribeygi et al., 2021). GLP-1R plays an important role in synaptic plasticity and memory formation, GLP-1R-knockout mice show cognitive impairment (Li et al., 2024).

Despite advancements, the precise molecular pathways mediating the neuroprotective effects of GLP-1RAs remain unclear. This review aims to elucidate T2DM related cognitive impairment mechanisms and propose potential mechanisms for GLP-1 cognitive enhancements.

## 2 T2DM causes changes in cognitive function

### 2.1 Cognitive impairment in T2DM: stages and risk factors

In individuals with T2DM, cognitive functioning undergoes distinct stages of impairment, ranging from diabetes-associated cognitive decrements to MCI and dementia (Biessels and Despa, 2018; Koekkoek et al., 2015). Numerous studies emphasize that diabetes independently poses a risk for cognitive dysfunction. For instance, blood sugar fluctuations in elderly diabetics were identified as a significant factor affecting white matter density (Tamura et al., 2017). Lower socioeconomic status correlated with an increased risk of early-onset dementia, particularly pronounced in T2DM patients (Li R. et al., 2023). Reviews underscore the chronic impact of T2DM on cognitive function and suggest a potential doubling of cognitive impairment incidence (Lyu et al., 2020). Moreover, diabetic patients with poor blood sugar control or cardiovascular disease face an elevated risk of cognitive impairment occurrence and progression (Dove et al., 2021; Kimura, 2016). Substantial evidence demonstrates the relationship between diabetes and cognitive dysfunction.

The intricate relationship between hyperglycemia, hyperinsulinemia, and the brain reveals substantial connections to cognitive decline (American Diabetes Association, 2021; Rawlings et al., 2014). Elevated glucose levels and disrupted glucose metabolism in diabetes and Alzheimer’s disease(AD) may lead to insulin resistance, oxidative stress, and inflammation, affecting cognitive levels (Wang Y. et al., 2023). Altered synaptic plasticity in the hippocampus, crucial for cognitive function, is associated with diabetes. Brain-Derived Neurotrophic Factor (BDNF), a neuropeptide integral to synaptic development, plays a key role in the central nervous system (Wang et al., 2022). Chaldakov et al. (2007) reported that the plasma BDNF levels of patients with T2DM were lower than those of healthy controls, and hyperglycemia could inhibit the output of BDNF from the brain. Ward et al. (2019) showed that diabetes could damage the integrity of the Neurovascular unit (NVU) in the hippocampus and reduce BDNF levels in endothelial cells and HT22 hippocampal neurons. Diabetes-induced reduction in BDNF secretion, along with increased receptor for advanced glycation end products (RAGE), contributes to cerebrovascular dysfunction and cognitive decline (Kim and Song, 2020).

### 2.2 Dopamine and cognitive function in diabetes

Dopamine (DA), a neurotransmitter known for its role in regulating behavior and movement, also modulates cognitive function. Interestingly, alterations in the dopaminergic system have been observed in DM. A comprehensive review highlighted the role of DA in cognitive function and emphasized DA dysfunction in DM. This finding supports the role of glucose toxicity in DM-related dopaminergic dysfunction and cognitive impairment, suggesting that advanced glycation end products (AGEs) and their precursor methylglyoxal (MGO) are linked to cognitive impairment and changes in the dopaminergic system. A longitudinal study further demonstrated that type 2 diabetes in older community-dwelling individuals is associated with a decline in verbal memory and fluency over approximately 5 years (Callisaya et al., 2019).

The effects of diabetes on brain atrophy may begin as early as midlife. However, a systematic review of observational studies showed substantial heterogeneity in the results (Kirvalidze et al., 2022). This heterogeneity precluded definitive conclusions regarding whether blood glucose levels are associated with cognition or dementia risk. Nonetheless, higher blood glucose levels were linked to greater amyloid burdens, brain atrophy, and reduced cortical thickness (Pignalosa et al., 2021).

### 2.3 Neuroinflammation and cognitive dysfunction in diabetes

Diabetes, as a chronic metabolic disorder, induces a chronic inflammatory state that damages the central nervous system. Neuroinflammation is a critical factor contributing to cognitive dysfunction and neurodegenerative diseases. Studies have shown that high glucose levels lead to the accumulation of lipid droplets in microglia and inhibit fat phagocytosis. These microglial defects in phagocytosis and pro-inflammatory factor secretion contribute to neurodegenerative changes and cognitive impairment. Inhibition of triggering receptor expressed on myeloid cells 1 (TREM1) has been shown to improve cognitive function in T2DM mice (Li Q. et al., 2023).

Animal studies have demonstrated that overexpression of triggering receptor expressed on myeloid cells 2 (TREM2) in the hippocampus can improve cognitive dysfunction caused by long-term high-fat diets (HFD). This improvement is associated with a significant increase in Iba-1/Arg-1 positive microglia, reduced neuroinflammation, and inhibition of microglial activation. These findings highlight the importance of TREM2 in promoting microglia polarization to the M2 anti-inflammatory phenotype and alleviating cognitive dysfunction (Wu M. et al., 2022).

### 2.4 Metabolic and mitochondrial dysfunction in diabetes

An animal experiment using db/db mice, a model of type 2 diabetes, demonstrated that disturbances in circulatory metabolism and brain energy metabolism, particularly mitochondrial dysfunction, may contribute to cognitive impairment. This study employed a multi-omics approach, integrating transcriptomic, metabolomic, and gut microbiota analyses, to identify key pathways and interactions underlying these metabolic disturbances (Song X. et al., 2022). Cerebral blood flow (CBF), which maintains proper cerebral perfusion and provides oxygen and energy substrates to neurons while removing metabolic waste, also plays a critical role in cognitive function (Fantini et al., 2016). A systematic review suggested that insufficient cerebral perfusion may be a potential cause of cognitive dysfunction (Wang Y. et al., 2021).

Platelets, fragments of megakaryocytes, contain abundant information related to the central nervous system. A comprehensive proteomic study of platelets in patients with T2DM and mild cognitive impairment (T2DM-MCI) versus those without (T2DM-nMCI) revealed that differentially expressed proteins are primarily involved in amyloidosis, mitochondrial autophagy, and insulin signaling pathways (Yu et al., 2021). This study provides valuable insights into the molecular mechanisms of cognitive impairment in T2DM.

Meta-analyses on gray and white matter changes in T2DM patients with cognitive impairment have concluded that T2DM impairs cognitive function by affecting specific brain structures (Ma et al., 2022). Additionally, the brain’s high energy demand makes it particularly dependent on mitochondrial function. A review highlighted that changes in lipid metabolism and mitochondrial dysfunction may be key factors contributing to cognitive dysfunction in diabetic patients (Potenza et al., 2021). During diabetes progression, the brain’s reliance on mitochondria makes it more vulnerable to oxidative damage compared to other regions.

**FIGURE 1:**
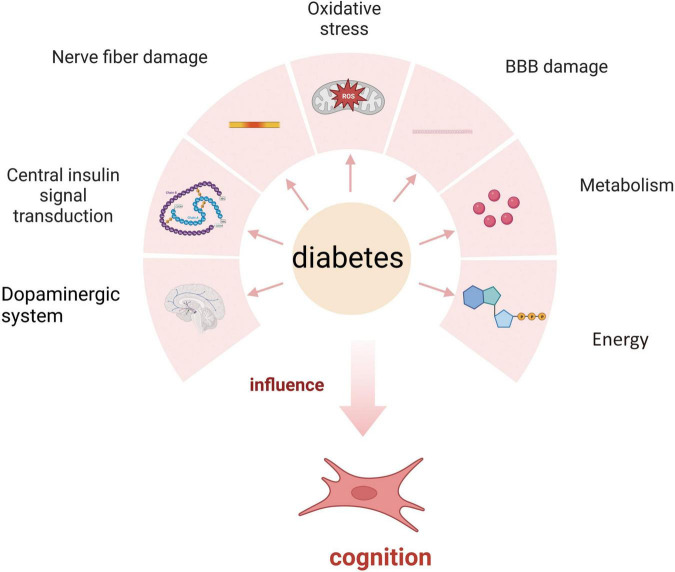
Mechanism of diabetes affecting cognition.

The relationship between diabetes and cognitive impairment involves many aspects, including central insulin signal transduction, nerve fiber damage caused by hyperglycemia, neuritis, oxidative stress, blood–brain barrier damage, vascular neuropathy, changes in the dopaminergic system, and the effects of advanced glycation end products on metabolism and energy (Figure 1). Researchers can develop screening or diagnostic tools and design targeted treatment and prevention strategies by elucidating the underlying mechanisms and exploring antidiabetic approaches to alleviate the burden of diabetes-related cognitive dysfunction.

## 3 The regulatory effect and mechanism of GLP-1 on cognitive impairment

### 3.1 GLP-1 improves cognitive function by affecting signal transduction pathways

GLP-1 enhances cognitive function through several key signal transduction pathways. Upon activation of the GLP-1 receptor, cAMP levels increase, which activates the cAMP response element-binding protein (CREB) (Bomba et al., 2018), This in turn induces the expression of brain-derived neurotrophic factor (BDNF), a critical neurotrophic factor that acts via its receptor, tropomyosin-related kinase B (TrkB). Binding of BDNF to TrkB activates intracellular signaling cascades, including the phosphorylation of CREB at its serine site, which is essential for neuroplasticity and memory formation (Kida and Serita, 2014). CREB activation is crucial not only for normal cognitive function but also for protecting against cognitive decline (Li et al., 2015). GLP-1 receptor analogs can also promote neuronal growth by enhancing CREB phosphorylation and increasing levels of p-ERK/ERK.

Furthermore, GLP-1R activation increases cAMP and alters downstream signaling pathways, including PKA, PI3K, PKC, and AKT, all of which contribute to its neuroprotective effects (Zhou et al., 2019). PI3K/Akt pathway also inhibits NF-κB inflammatory signaling (Kopp et al., 2022). Zheng et al. (2021) found that GLP-1 could regulate glycolysis in astrocytes through the PI3K/Akt pathway, which exerts neuroprotective effects against AD. One important pathway involved is the PI3K/AKT signaling cascade, which has been shown to reduce tau hyperphosphorylation and neurofilament protein accumulation, thereby protecting against memory impairment (Wang Y. et al., 2023).

When GLP-1 binds to its receptor, it activates Akt, which subsequently inhibits GSK3β and reduces the accumulation of harmful proteins, such as α-synuclein. Elevated cAMP activates multiple downstream pathways that help reduce inflammation, oxidative stress, and apoptosis. GLP-1R activation has been shown to downregulate proapoptotic proteins such as caspase-3, caspase-9, Bax, and Bcl-2. Moreover, studies have demonstrated that GLP-1 suppresses apoptotic cell death via the PI3K/Akt/mTOR/GCLc/redox signaling pathway in neuronal cultures (Nagata, 2018). Chen et al. (2016) reported that GLP-1R induction downregulated proapoptotic elements such as caspase-3, caspase-9, Bax and Bcl-2 in the neurons of mice. Furthermore, GLP-1 activation enhances mitochondrial function through AMPK-mediated phosphorylation, which is essential for cellular energy metabolism and neuroprotection (Kimura et al., 2009).

Some studies have shown that in GLP-1R-overexpressing cells, PKA and AMPK are activated as the main downstream signaling molecules of GLP-1 (9-36), which is helpful for restoring cell vitality to meet the challenge of glutamic acid. In addition, when PKA and AMPK are inhibited in a dose-dependent manner, the cytotoxicity of glutamic acid is aggravated (Li Y. et al., 2021). Research indicated that Exendin-4 treatment increased cAMP levels, reduced Na^+^/K^+^ ATPase activity, and modulated the GLP-1 receptor in the rat choroid plexus *in vitro* (Botfield et al., 2017).

Recent findings also suggest that GLP-1 modulates the SIRT1 pathway, which plays a crucial role in regulating inflammation and autophagy. Activation of GLP-1R has been shown to activate SIRT1, promoting neuroprotective effects through the regulation of autophagy and reduction of inflammation. This suggests that GLP-1 may be a promising therapeutic target for neurodegenerative diseases such as Alzheimer’s (Wu L. et al., 2022).

Current research also confirmed the possible interaction among AMPK/SIRT1, autophagy and NLRP3 inflammatory corpuscle pathway and their role in the potential anti-inflammatory neuroprotective effect of Liraglutide (Ammar et al., 2022). A study indicated that semaglutide could regulate the expression of GLUT4 to mediate glucose transport through SIRT1, thereby improving glucose metabolism dysfunction in AD mice and cells (Wang Z. et al., 2023). Studies had shown that liraglutide has anti-inflammatory and anti-demyelination effects on experimental autoimmune encephalitis (EAE) mice, which is related to the regulation of the p-AMPK pathway, autophagy and the NLRP3 pathway (Song S. et al., 2022).

**FIGURE 2:**
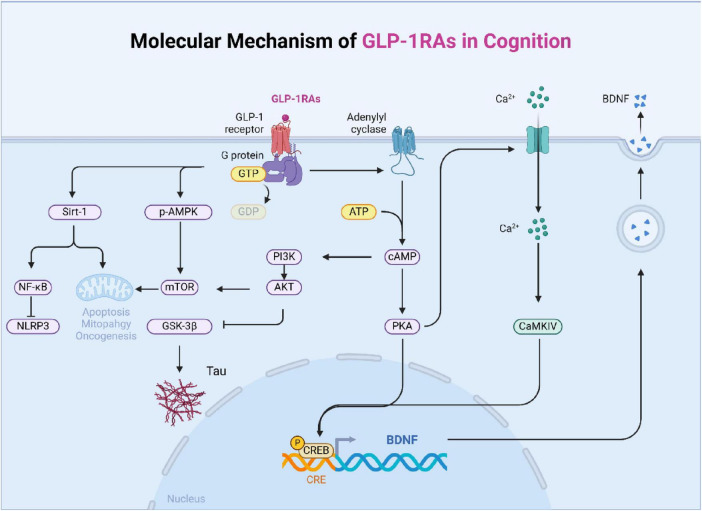
The main signal pathway of GLP-1 affecting cognition. The mechanism of diabetes affecting cognition is complex and interrelated, which is mainly divided into six categories: changes in dopaminergic system, central insulin signal transduction, nerve fiber damage, oxidative stress, blood-brain barrier damage, and changes in energy and metabolism.

In conclusion, GLP-1 improves cognitive function by engaging multiple signaling pathways, including CREB, PI3K/Akt, mTOR, and SIRT1. These pathways regulate various aspects of neuronal health, such as neuroplasticity, apoptosis, mitochondrial function, and inflammation, underscoring the potential of GLP-1 in treating cognitive disorders (Figure 2).

### 3.2 GLP-1 improves cognition by regulating the oxidative metabolic pathway of cells

Oxidative stress refers to an imbalance in free radicals and the cellular antioxidant defense system in favor of the formation of free radicals (Yaribeygi et al., 2020b; Yaribeygi et al., 2021). Harmful oxidative stress is closely involved in many complications, as well as neuronal problems and cognitive dysfunction (Jurcau and Simion, 2020; Yaribeygi et al., 2020a). GLP-1 exerts antioxidant effects on various tissues and the central nervous system. Li et al. (2016) found that the GLP-1RAs exendin-4 and liraglutide could improve oxidative stress and cognitive dysfunction caused by middle cerebral artery occlusion in diabetic mice and could reduce the production of reactive oxygen species and the expression of related inflammatory proteins in the brain and blood. Zheng et al. (2021) found that GLP-1 could improve the cognition of mice by enhancing aerobic glycolysis and reducing the levels of oxidative phosphorylation and oxidative stress in the brain. Aerobic glycolysis is thought to exert protective effects by providing lactic acid to neurons with insufficient energy. Evidence also shows that lactic acid has beneficial effects on stressed neurons. Lactic acid supplementation has been proven to reduce brain damage in hypoxic-ischemic encephalopathy (Roumes et al., 2021). Increased lactic acid concentrations in the hippocampus improve nerve injury and relieve depression (Carrard et al., 2018). It has also been reported that exercise can improve cognition by producing lactic acid, which can cross the blood–brain barrier and promote the production of BDNF (El Hayek et al., 2019). In a 26-week randomized, double-blind, placebo-controlled Danish population study, patients with an average age of 66 years were included. Compared with that in the placebo group, liraglutide improved glucose metabolism and cognitive function in patients with MCI and AD. A study confirmed that liraglutide could prevent the decrease in glucose metabolism in patients with AD and suggested that liraglutide could ameliorate cognitive impairment, synaptic dysfunction and disease development (Li et al., 2024).

### 3.3 GLP-1 can reduce the level of pathological markers of AD

AD is a progressive and irreversible neurodegenerative disease, and its etiology and pathogenesis are still unclear. Approximately 6%–8% of people over 65 years of age have AD; it is known that this risk increases with age and is more common in women (1.5 times that of men) (McClean et al., 2011; Zhao et al., 2021). In an AD mouse model, GLP-1RA has been shown to reduce pathological markers of AD, including amyloid oligomers and plaque load. It also reduces glial cell activation and improves memory (Kim et al., 2009; McClean and Hölscher, 2014; McClean et al., 2011; Zhao et al., 2021). A study showed that liraglutide significantly improved memory retention in SAMP8 mice (Senescence-Accelerated Mouse Prone 8, which are widely recognized model for age-related cognitive decline and neurodegenerative diseases). It also increased the total number of hippocampal CA1 pyramidal neurons compared to age-matched controls (Hansen et al., 2015). In AD transgenic mice, systemic administration of liraglutide for 8 weeks could prevent memory impairment, neuronal loss and deterioration of synaptic plasticity in the hippocampus. In addition, as shown by the number of activated glial cells, liraglutide can significantly reduce the deposition and inflammation of amyloid plaques (Wiciñski et al., 2019; Zhao et al., 2021). A study showed that the treatment of exenatide could strongly reverse the changes of transcription group of brain endothelial cells and the infiltration of blood-brain barrier in aging mice (Zhao et al., 2020). A randomized controlled trial showed that GLP-1RAs prevents the decline of cerebral metabolic rate for glucose (CMRglc) in AD, and GLP-1RAs may increase the expression of glucose transporters (e.g., GLUT1) at the blood-brain barrier (BBB) (Gejl et al., 2017). These can be seen that GLP-1RAs has a promising therapeutic effect on AD. However, some experiments showed that the improvement of GLP-1RAs on AD was not clear. In addition to a decrease in Aβ42 in extracellular vesicles (EV), compared to placebo, exenatide showed no significant difference or trend in cognitive function, MRI cortical thickness, volume, or biomarkers in cerebrospinal fluid, plasma, or EVs (Mullins et al., 2019).

### 3.4 GLP-1 improves cognition by protecting neurons

Some recent studies have shown that GLP-1RAs acted on the nervous system and provides neuroprotection. In an experiment where neurons were treated with GLP-1, it was observed that the calcium response to glutamate and membrane depolarization was attenuated. GLP-1 plays a crucial role in regulating neuronal plasticity and survival by modulating the calcium response to glutamate and membrane depolarization (Gilman et al., 2003). It has been reported that a new GLP-1 analog, Val(8) GLP-1-Glu-PAL, can promote hippocampal neuron formation. GLP-1 can effectively alleviate the symptoms of AD, which may be related to increasing the level of growth factors that protect the function of nerve cells in the brain, reducing the level of chronic inflammation and oxidative stress in the brain, and alleviating the rate of nerve loss (Tai et al., 2018; Zhao et al., 2021). Data indicate that GLP-1 receptor mimetics exert anti-inflammatory effects on neuronal structures (Hölscher, 2014; Yoon et al., 2020). Increasing evidence shows that GLP-1 analogs have neuroprotective effects in animal models. GLP-1R-deficient mice exhibit learning disabilities, and this can be reversed by introducing the GLP-1R gene into the hippocampus. GLP-1R-knockout mice show synaptic plasticity and memory formation disorders (Abbas et al., 2009). An experimental study showed that diabetes increases GLP-1 receptor expression and receptor nitration in human brain microvascular pericytes (HBMPCs). Stimulating HBMPCs with exendin-4 improved vascular inflammation and oxidative stress caused by diabetes, and also enhanced the function of peripheral cells to some extent (Bailey et al., 2022). In an animal experiment, mice treated with liraglutide for 8 weeks showed that the abnormality of insulin receptor was significantly reduced and the load of amyloid plaque was also reduced (Long-Smith et al., 2013). A study showed that Semaglutide had anti-inflammatory and anti-apoptotic effects. It attenuates the inflammatory response and LDH release induced by LPS and nigericin by blocking NLRP3 inflammasome activation. Additionally, semaglutide reduced seizure severity in pentylenetetrazole (PTZ) kindled mice (Wang L. et al., 2021). A study indicated that tirzepatide ameliorates high glucose-induced damage to nerve cells (Fontanella et al., 2024). It regulates neuronal growth (CREB/BDNF), anti-apoptosis (BAX/Bcl-2), neuronal differentiation (pAkt, MAP2, GAP43, and AGBL4), and neuronal glucose homeostasis (GLUT1, GLUT3, and GLUT4), thereby alleviating neurodegeneration. A study indicated that exenatide alleviates the reduction in local motor activity and anxiety-like behavior in mice caused by a HFD (Lin et al., 2023). HFD led to astrocyte proliferation, microglial activation, and upregulation of IL-1β, IL-6, and TNF-α in the hippocampus and cortex, while exenatide treatment significantly reduced these inflammatory markers. Furthermore, exenatide enhanced the expression of phosphorylated ERK and M2-type microglial markers, demonstrating its neuroprotective and anti-inflammatory effects.

## 4 Clinical trials and efficacy of GLP-1RAs

GLP-1RAs are widely used in clinical practice, with benefits that extend beyond blood sugar control. They also demonstrate advantages in areas such as weight loss, improved cardiovascular health, reduced risk of cardiovascular events, and potential cognitive benefits. These characteristics make GLP-1RAs important treatment options for type 2 diabetes and obesity. We will outline some common GLP-1RAs, including liraglutide, exenatide, dulaglutide, and lixisenatide, along with their efficacy and limitations regarding cognitive function as observed in clinical trials (Table 1).

**TABLE 1:** Efficacy of GLP-1 receptor agonists (GLP-1RAs) in clinical trials.

Study	No. of participants	Type of participants	Generic name of GLP-1RAs	Study design	Main finding	Ethics approval number
Meissner et al., 2024	156	Early Parkinson’s disease	Lixisenatide	Double-blind, randomized, placebo-controlled trial, phase II study	The improvement in motor symptom progression in early PD patients was less than that in the placebo group, and adverse reactions were observed.	NCT03439943
Klausen et al., 2022	127	Alcohol use disorder (AUD)	Exenatide	Randomized, placebo-controlled, double-blinded clinical trial	Exenatide significantly attenuated fMRI alcohol cue reactivity in the ventral striatum and septal area.	NCT03232112
Cukierman-Yaffe et al., 2020	9901	Aged =50 years withT2DM, HbA1c =9.5% on a maximum of two oral glucose-lowering drugs with or without basal insulin, and a BMI=23 kg/m^2^	Dulaglutide	Randomized, double-blind placebo-controlled trial	The risk of significant cognitive impairment was reduced by 14% in patients assigned to dulaglutide.	NCT01394952
Aviles-Olmos et al., 2013	45	patients with moderate Parkinson’s disease (PD)	Exenatide	Randomized, single-blind trial	The exenatide group showed clinically relevant improvements in PD in both motor and cognitive measures compared to the control group.	NCT01174810
Li Q. et al., 2021	50	patients with T2DM	Liraglutide	Prospective, parallel assignment, open-label, phase III study	Liraglutide slows cognitive decline in patients with type 2 diabetes mellitus.	NCT03707171
Cheng et al., 2022	36	Patients withT2DM and inadequately controlled with metformin monotherapy	Liraglutide	Randomized, open-label, parallel-group study	Liraglutide enhanced impaired brain activation and restored cognitive domains in patients with type 2 diabetes, while dapagliflozin and acarbose did not demonstrate these effects.	NCT03961659
Ishøy et al., 2017	40	Patients with schizophrenia spectrum disorder who are obese and treated with antipsychotics	Exenatide	Investigator-initiated, double-blind, randomized, placebo-controlled trial	The non-metabolic effects of exenatide in patients with schizophrenia were not significant.	NCT01794429
Farr et al., 2016	20	Patients with T2DM	Liraglutide	Randomized, placebo-controlled, double-blind	Liraglutide altered circulating hormone levels important for energy homeostasis, affecting CNS food cue perception. This may have led to compensatory changes in energy balance, limiting liraglutide’s long-term efficacy in reducing body weight.	NCT01562678
Zhang et al., 2019	20	Patients with diabetes and inadequate glycemic control with metformin monotherapy	Liraglutide or exenatide	Randomized	GLP-1Ra treatment significantly improved MoCA and olfactory test total score and increased odor-induced brain activation in obese subjects with diabetes	NCT02738671
Dei Cas et al., 2024	32	Patients aged 50–80 years, stable medications for the past 3 months and diagnosis of MCI	Exenatide	Randomized (1:1), open-label, controlled proof-of-concept study	In patients with MCI, a 32-week trial with slow-release exenatide had no beneficial effect on cognitive performance.	NCT03881371
Vadini et al., 2020	40	Metformin-treated obese subjects with T2DM	Liraglutide	A longitudinal, randomized, controlled, parallel-arm study	Liraglutide may slow memory decline in diabetic patients during early, possibly preclinical stages of the disease.	Not reported.
Bae et al., 2020	29	Individuals with T2DM,14 lean and 15 obesity	Lixisenatide	Randomized, single-blinded, crossover study	Acute administration of lixisenatide differentially affected functional brain activation in these individuals, especially in those who decreased their caloric intake after lixisenatide injection.	NCT02745470
Gejl et al., 2017	38	Patients with AD, recruited from dementia clinics in Central Denmark	Liraglutide	Randomized, placebo-controlled, double-blinded intervention study	The restoration of brain glucose availability and neuronal metabolism with GLP-1 or its analogs may protect against cognitive impairment in Alzheimer’s disease.	NCT01469351

In a 12-month, two-phase trial, subcutaneously administered lixisenatide moderately reduced the progression of motor dysfunction in patients with early Parkinson’s disease (PD) compared to placebo (Meissner et al., 2024). However, it had gastrointestinal side effects, with 46% of subjects experiencing nausea and 13% experiencing vomiting. Alcohol use disorder (AUD) is a chronic and relapsing brain disorder. A study indicated that exenatide significantly reduced fMRI alcohol cue reactivity in key brain regions, such as the ventral striatum and septal area, which are important for drug reward and addiction-regions with high GLP-1 receptor expression. Additionally, findings suggested that DA transporter availability was lower in the exenatide group compared to the placebo group, potentially indicating altered DA dynamics associated with reduced cravings and cue sensitivity (Klausen et al., 2022). These findings highlight the potential of GLP-1RAs in treating AUD. Additionally, a clinical study suggested that the exenatide group showed clinically relevant improvements in PD across both motor and cognitive measures compared to the control group (Aviles-Olmos et al., 2013). A REWIND study on dulaglutide indicated that long-term treatment with this medication may help reduce cognitive impairment in individuals with type 2 diabetes (Cukierman-Yaffe et al., 2020). A prospective clinical trial indicated that, compared with the control group, liraglutide significantly increased activation in the dorsolateral prefrontal cortex and orbitofrontal cortex regions of the brain. After liraglutide treatment, cognitive scores were significantly correlated with changes in these activating brain regions (Li Q. et al., 2021). A head-to-head study indicated that liraglutide enhanced impaired brain activation and restored cognitive domains in patients with type 2 diabetes, while dapagliflozin and acarbose did not demonstrate these effects, highlighting the superiority of GLP-1RAs in cognitive function (Cheng et al., 2022). In a preliminary clinical trial, the results of exenatide’s non-metabolic effects in patients with schizophrenia were non-significant, which indicated challenges in translating the cognitive-enhancing effects observed in animal studies to humans (Ishøy et al., 2017). A study indicated that liraglutide use could alter Glucose-dependent insulinotropic polypeptide (GIP) and leptin levels. Increased GIP may promote the anorexigenic effects of liraglutide, while decreased leptin might counteract these effects. These findings suggested that liraglutide could alter the circulating levels of hormones important for energy homeostasis, which in turn influenced the central nerve system (CNS) perception of food cues. This might have also limited the effectiveness of long-term weight loss with liraglutide (Farr et al., 2016). A study indicated that after 3 months of GLP-1 receptor agonist (GLP-1Ra) treatment, obese subjects with diabetes showed improved Montreal Cognitive Assessment (MoCA) scores, higher olfactory test scores, and enhanced activation of the right parahippocampus in response to odors (Zhang et al., 2019). A clinical trial found that in patients with MCI, a 32-week trial of slow-release exenatide had no beneficial effect on cognitive performance (Dei Cas et al., 2024). A study indicated that liraglutide may slow the decline of memory function in diabetic patients in the early and possibly preclinical stages of the disease (Vadini et al., 2020). Additionally, they found that only in the liraglutide arm, the time to weight loss (reflecting the duration of drug exposure) was directly related to the improvement in both short-term memory and the memory domain z-score. The study by Bae et al. (2020) indicates that brain responses to visual food cues differ between lean and obese individuals with T2DM. Lixisenatide injection significantly reduced the functional activation of the fusiform gyrus and lateral ventricle in obese individuals with T2DM compared to lean individuals with T2DM (Bae et al., 2020). The study by Gejl et al. (2017) indicated that, compared to the placebo group, liraglutide treatment significantly increased the blood-brain glucose transfer capacity (T max) in the cerebral cortex. They suggest that the restoration of brain glucose availability and neuronal metabolism with GLP-1 or its analogs may potentially protect against cognitive impairment in AD. Regarding the relationship between GLP-1RAs and cognitive function, the conclusions drawn from these studies are varied and inconsistent. While some studies indicate that GLP-1RAs may improve cognitive function or reduce cognitive decline, other studies suggest that their effects are limited or not statistically significant. This variability may be related to differences in study design, participant characteristics, and measurement methods. Overall, more research is needed to fully understand the relationship between GLP-1RAs and cognitive outcomes.

## 5 Discussion

Our analysis of the potential mechanisms through which GLP-1 improves cognitive function in diabetes highlights their interdependence and potential synergies. For instance, GLP-1’s activation of signal transduction pathways not only reduces oxidative stress and apoptosis but also mitigates neuroinflammation and neurotoxicity. However, the exact mechanisms underlying these effects remain unclear, particularly whether the cognitive improvements are a direct result of GLP-1 action or are mediated indirectly via enhanced glucose regulation.

One limitation of the current literature is the lack of extensive clinical studies exploring the direct neuroprotective effects of GLP-1RAs in human populations. Although preclinical studies have demonstrated promising outcomes, more randomized controlled trials are needed to assess whether GLP-1 can consistently improve cognitive function in patients with diabetes or those at risk of neurodegenerative conditions. Additionally, the potential long-term benefits of GLP-1 therapy on different stages of cognitive impairment, such as MCI and dementia, warrant further exploration.

Despite the extensive evidence supporting the beneficial effects of GLP-1RAs on the central nervous system (CNS), emerging studies have raised concerns about potential adverse effects. Drucker (2024) highlighted that while GLP-1RAs demonstrate significant cardiorenal and metabolic benefits, their widespread use has also been associated with certain psychiatric adverse events, such as nervousness, stress, and eating disorders. These findings suggest that GLP-1 signaling may, under certain conditions, contribute to neuronal stress or dysregulation of emotional and behavioral responses. Similarly, Chen et al. (2024) reported that prolonged activation of GLP-1 receptors could lead to psychiatric adverse events (AEs), including sleep disorders and fear of injection, particularly in vulnerable populations. Although these adverse effects are less frequently reported and require further validation, they underscore the need for a more comprehensive understanding of the potential risks associated with GLP-1RAs. Future studies should aim to elucidate the mechanisms underlying these contradictory observations and identify the specific conditions under which GLP-1RAs might exert detrimental effects on the CNS. Clinicians should remain vigilant for potential psychiatric AEs in patients receiving GLP-1RA therapy and consider early intervention strategies to optimize risk management. Additionally, future research should focus on distinguishing between the direct neuroprotective effects of GLP-1 and its indirect benefits resulting from improved metabolic control. This will require experimental designs that isolate glucose-independent pathways, such as by controlling for glucose levels in preclinical and clinical studies. Furthermore, investigating patient-specific factors, including genetic predispositions and disease progression variability, will be critical for understanding the full therapeutic potential of GLP-1RAs and optimizing personalized treatment strategies for diabetes-related cognitive dysfunction.

## 6 Conclusion

In conclusion, GLP-1, initially identified as an intestinal insulin-secreting molecule, exhibits multifaceted effects beyond blood sugar control, showing promise in enhancing cognitive function. While our findings support the potential of GLP-1RAs in improving cognitive function, there is a need for further clinical investigations to validate these mechanisms and to explore the long-term effects of GLP-1-based therapies in human populations. Expanding research in this area will be crucial for developing targeted treatments to mitigate the burden of cognitive impairment in diabetes.
